# High Interstitial Fluid Pressure Is Associated with Tumor-Line Specific Vascular Abnormalities in Human Melanoma Xenografts

**DOI:** 10.1371/journal.pone.0040006

**Published:** 2012-06-29

**Authors:** Trude G. Simonsen, Jon-Vidar Gaustad, Marit N. Leinaas, Einar K. Rofstad

**Affiliations:** Group of Radiation Biology and Tumor Physiology, Department of Radiation Biology, Institute for Cancer Research, Oslo University Hospital, Oslo, Norway; Virginia Commonwealth University, United States of America

## Abstract

**Purpose:**

Interstitial fluid pressure (IFP) is highly elevated in many solid tumors. High IFP has been associated with low radiocurability and high metastatic frequency in human melanoma xenografts and with poor survival after radiation therapy in cervical cancer patients. Abnormalities in tumor vascular networks have been identified as an important cause of elevated tumor IFP. The aim of this study was to investigate the relationship between tumor IFP and the functional and morphological properties of tumor vascular networks.

**Materials and Methods:**

A-07-GFP and R-18-GFP human melanomas growing in dorsal window chambers in BALB/c *nu/nu* mice were used as preclinical tumor models. Functional and morphological parameters of the vascular network were assessed from first-pass imaging movies and vascular maps recorded after intravenous bolus injection of 155-kDa tetramethylrhodamine isothiocyanate-labeled dextran. IFP was measured in the center of the tumors using a Millar catheter. Angiogenic profiles of A-07-GFP and R-18-GFP cells were obtained with a quantitative PCR array.

**Results:**

High IFP was associated with low growth rate and low vascular density in A-07-GFP tumors, and with high growth rate and high vascular density in R-18-GFP tumors. A-07-GFP tumors showed chaotic and highly disorganized vascular networks, while R-18-GFP tumors showed more organized vascular networks with supplying arterioles in the tumor center and draining venules in the tumor periphery. Furthermore, A-07-GFP and R-18-GFP cells differed substantially in angiogenic profiles. A-07-GFP tumors with high IFP showed high geometric resistance to blood flow due to high vessel tortuosity. R-18-GFP tumors with high IFP showed high geometric resistance to blood flow due to a large number of narrow tumor capillaries.

**Conclusions:**

High IFP in A-07-GFP and R-18-GFP human melanoma xenografts was primarily a consequence of high blood flow resistance caused by tumor-line specific vascular abnormalities.

## Introduction

While the interstitial fluid pressure (IFP) in normal tissues is actively controlled and remains close to atmospheric levels, IFP in most human tumors is highly elevated [1]. Measurements of IFP in a wide range of tumor types have given typical IFP values in the range of 10 to 40 mmHg [2]–[4], and IFP values as high as 100 mmHg have been measured in subcutaneous nodules in melanoma patients [5]. Theoretical and experimental studies have identified elevated IFP as an important barrier to drug delivery and, particularly, the delivery of monoclonal antibodies and other macromolecular drugs that depend on transport by convection rather than diffusion [6]–[8]. Furthermore, high IFP in the primary tumor has been shown to predict poor survival after radiation therapy in cervical cancer patients independent of tumor oxygenation and other prognostic factors [9], [10]. Preclinical studies of human melanoma xenografts in our lab have shown an association between high IFP and low radiocurability through both hypoxia-dependent and hypoxia-independent mechanisms [11], [12]. Moreover, high IFP was associated with high incidence of pulmonary and lymph node metastasis in small tumors without hypoxic regions [13].

The elevated IFP in solid tumors is a consequence of increased transcapillary fluid flow, increased resistance to interstitial fluid flow, and impaired lymphatic drainage [1], [6]. Increased transcapillary fluid flow results from severe structural and functional abnormalities in the microvasculature of tumors [14]–[16]. Tumor vessels show high permeability to fluid and macromolecules as a result of incomplete endothelial lining, discontinuous or absent basement membranes, and lack of pericyte coverage [14]. Furthermore, tumor vascular networks are characterized by tortuous and elongated vessels, arterio-venous shunts, blind ends, excessive branching, increased intervessel distances, and loss of vessel hierarchy [15], [16]. Consequently, these networks show high geometric resistance to blood flow, which increases the hydrostatic pressure in the tumor vessels and drives the flow of fluid from the vascular to the interstitial space [17], [18]. Effective transport of this fluid through the interstitial space is hindered by interstitial abnormalities, including high cell densities and increased stiffness of the extracellular matrix [6]. Furthermore, most solid tumors lack functional lymphatics within the tumor compartment [1], [19]. Drainage of excess fluid from the tumor interstitium is thus impaired. The net result is accumulation of fluid and, hence, increased fluid pressure in the interstitial space. In experimental tumors, IFP has been found to be uniformly elevated throughout the tumor and to drop steeply towards atmospheric levels in the tumor periphery [13], [20].

IFP has been shown to vary widely among individual human tumors of the same histological type [5], and among xenografted tumors of the same tumor line [13]. Moreover, it has been shown experimentally that the increase in tumor IFP occurs early in tumor development and in close association with tumor angiogenesis [21]. Physiological angiogenesis is a tightly regulated process controlled by the balance and interplay of several pro- and anti-angiogenic factors. Tumors induce angiogenesis by upregulating pro-angiogenic factors, and by downregulating anti-angiogenic factors, thereby tipping the balance in favor of the pro-angiogenic factors [22], [23]. The uncontrolled and stochastic nature of the angiogenic process in tumors leads to the structural and functional abnormalities characteristic of tumor vascular networks and, furthermore, it leads to large intertumor heterogeneity in vascular parameters [16]. The large intertumor heterogeneity in IFP observed in tumors of the same histological type is therefore believed to reflect intertumor heterogeneity in vascular permeability and/or blood flow resistance [1]. As most tumors show high vascular permeability, the microvascular pressure has been proposed to be the principle driving force for elevated IFP in tumors [18], [24]. Thus, as fluid can move relatively freely between the vascular and interstitial space, any gradient in fluid pressure across the vessel wall is quickly neutralized. Consequently, an increase in microvascular pressure as a result of high blood flow resistance will be followed by a corresponding increase in IFP.

Lowering of tumor IFP has the potential of improving patient outcome both by increasing the effect of conventional therapies and by reducing the risk of distant metastasis. Several strategies to lower tumor IFP are being investigated [25]–[30]. As a result of the close relationship between IFP and tumor angiogenesis, many of these strategies are directed towards the tumor vasculature. One such strategy is anti-angiogenic therapy. Treatment with agents that block the function of pro-angiogenic factors has been proposed as a strategy to transiently restore the balance between pro- and anti-angiogenic factors and, thus, normalize the tumor vasculature [27]. Vascular normalization could potentially reduce IFP by reducing vascular permeability and/or by reducing the blood flow resistance. Consequently, a decrease in IFP after anti-angiogenic therapy has been demonstrated in both experimental [28], [29] and human tumors [30]. The underlying mechanisms are however not well understood.

Even though the microvascular pressure is believed to be the principle driving force for elevated IFP in solid tumors, few experimental studies have investigated in detail the relationship between vascular morphology, vascular function, and elevated IFP. Intravital microscopy provides detailed information on both the functional and structural properties of tumor vascular networks, and is thus a well-suited method to study vascular abnormalities causing elevated IFP. In the current study, xenografted A-07-GFP and R-18-GFP human melanomas were subjected to intravital microscopy and subsequent IFP measurement. A detailed analysis of vascular morphology and function showed that high IFP was associated with high blood flow resistance caused by tumor-line specific vascular abnormalities.

## Materials and Methods

### Ethics statement

All animal experiments were approved by The Norwegian Animal Research Authority (approval number: FOTS-2751) and were performed according to the Interdisciplinary Principles and Guidelines for the Use of Animals in Research, Marketing, and Education (New York Academy of Sciences, New York, NY).

### Mice

Adult (8–120 weeks of age) female BALB/c *nu/nu* mice were used as host animals for dorsal window chamber preparations. The mice were bred at our institute and maintained under specific pathogen-free conditions at constant temperature (24–126°C) and humidity (30–120%). Sterilized food and tap water were given *ad libitum*.

### Cells and Multicellular Spheroids

A-07 and R-18 human melanoma cells [31] were constitutively transfected with green fluorescent protein (GFP) by lipofection. The transfected cells (A-07-GFP and R-18-GFP) were grown as monolayers in RPMI 1640 (25 mM HEPES and l-glutamine) supplemented with 13% bovine calf serum, 250 µg/ml penicillin, 50 µg/ml streptomycin, and 700 µg/ml (A-07-GFP) or 2200 µg/ml (R-18-GFP) genetecin. Multicellular spheroids were produced and maintained by using a liquid-overlay culture technique [32]. Cell and spheroid cultures were incubated at 37°C in a humidified atmosphere of 5% CO_2_ in air and subcultured twice a week.

### Anesthesia

Window chamber implantation, intravital microscopy, and IFP measurements were carried out with anesthetized mice. Fentanyl citrate (Janssen Pharmaceutica, Beerse, Belgium), fluanisone (Janssen Pharmaceutica), and midazolam (Hoffmann-La Roche, Basel, Switzerland) were administered intraperitoneally (i.p.) in doses of 0.63 mg/kg, 20 mg/kg, and 10 mg/kg, respectively. After surgery, the mice were given buprenorphine (Temgesic; Schering-Plough, Brussels, Belgium) i.p. in a dose of 0.12 mg/kg. The body core temperature of the mice was maintained at 37–128°C by using a hot air generator during intravital microscopy and a heating pad during window chamber implantation and IFP measurements.

### Window Chamber Preparations

Window chambers were implanted into the dorsal skin fold as described previously [33]. Briefly, the chamber consisted of two parallel frames that sandwiched an extended double layer of skin. Before the chamber was implanted, a circular hole with a diameter of ∼6.0 mm was made in one of the skin layers. A plastic window with a diameter of 6.0 mm was attached to the frame on the surgical side with a clip to provide visual access to the fascial side of the opposite skin layer. Tumors were initiated by implanting a spheroid or a tumor fragment with a diameter of 200–1200 µm directly onto the fascial side of the intact skin layer. After chamber implantation, the mice were kept at 32°C and 50–120% humidity.

### Intravital Microscopy

Imaging was performed with an inverted fluorescence microscope equipped with filters for green and red light (IX-71; Olympus, Munich, Germany), a black and white CCD camera (C9300-024; Hamamatsu Photonics, Hamamatsu, Japan), and appropriate image acquisition software (Wasabi; Hamamatsu Photonics). During imaging, the mice were kept in a specially constructed holder that fixed the window chamber to the microscope stage. Tetramethylrhodamine isothiocyanate-labeled (TRITC)-dextran with molecular weight of 155 kDa (Sigma Aldrich, St. Louis, MO) was used as a vascular tracer. A first-pass imaging movie was recorded after injection of a 0.2 ml bolus of TRITC-dextran (50 mg/ml) into the lateral tail vein. The movie was recorded at a frame rate of 22.3 fps by use of a ×2 objective lens, resulting in a time resolution of 44.8 ms, a field of view of 5.97×5.97 mm^2^, and a pixel size of 7.46×7.46 µm^2^. For analysis of vascular morphology, the tumor vasculature was mapped by recording 1 to 4 single frames with a ×4 objective lens, resulting in a field of view of 3.80×3.80 mm^2^ and a pixel size of 3.71×3.71 µm^2^. All recordings were stored and analyzed offline.

### Analysis of Vascular Morphology

Two-dimensional projected vascular masks were established from stored images both manually and by using an in-house made computer program. The algorithms used for identification of microvascular networks were implemented in MATLAB software (The MathWorks, Natick, MA) as previously described [33]. The following morphological parameters were calculated from masks obtained from high-magnification images recorded with a ×4 objective lens: vascular area fraction (# pixels in the vascular mask/# tumor pixels), total vessel density (total vessel length per tumor area), density of large vessels (length of vessels with diameter >20 µm per tumor area), and median vessel diameter. Vessel segment length (i.e. the distance between branching points along a vessel) and vessel tortuosity were measured in high-magnification transillumination images. Vessel tortuosity (*T*) was defined as T (%)  =  (SL-L) *100/SL, where *SL* represents the distance between branching points along a vessel, and *L* represents the distance between the branching points along a straight line (i.e. the shortest possible distance between the branching points). This definition is the same as that previously used by Tozer et al [34]. Median vessel segment length and median vessel tortuosity were calculated from 100–1250 randomly selected vessels in each tumor.

### Analysis of Vascular Function

Blood supply time (BST) images were produced by assigning a BST value to each pixel of vascular masks established from the movies. The BST of a pixel was defined as the time difference between the frame showing maximum fluorescence intensity in the pixel and the frame showing maximum fluorescence intensity in the main supplying artery. This method has previously been described in detail [35], [36]. Two R-18-GFP tumors were excluded from BST analysis because of noise in the intensity curves. Plasma velocities in tumor arterioles (TAs) and tumor venules (TVs) were calculated from the time lag of the peak fluorescence intensity along individual vessel segments [36]. Mean TA plasma velocity and mean TV plasma velocity were calculated from 3–12 TAs and 3–12 TVs in each tumor.

### IFP Measurements

IFP was measured in the center of the tumors immediately after intravital microscopy imaging by using a Millar SPC 320 catheter equipped with a 2F Micro-Tip transducer with diameter 0.66 mm (Millar Instruments, Houston, TX) [37]. The catheter was inserted into the tumor without removing the window, and was connected to a computer via a Millar TC-510 control unit and a model 13-66150-50 preamplifier (Gould Instruments, Cleveland, OH). Data acquisition was carried out by using LabVIEW software (National Instruments, Austin, TX).

### Immunohistochemical Assessment of Hypoxia

Pimonidazole hydrochloride, dissolved in 0.9% sodium chloride, was administered i.p. in doses of 30 mg/kg. The tumors were resected approximately 4 h after pimonidazole administration and fixed in phosphate-buffered 4% paraformaldehyde. A peroxidase-based immunohistochemical assay was used to detect tumor hypoxia [38]. Histological preparations were incubated with polyclonal rabbit antiserum to pimonidazole-protein adducts. Diaminobenzidine was used as chromogen, and hematoxylin was used for counterstaining.

### RNA Isolation and cDNA Synthesis

Total RNA was isolated from cultured A-07-GFP and R-18-GFP cells in exponential growth. The RNeasy Mini Kit (Qiagen, Hilden, Germany) was used according to the manufacturer’s instructions. Possible genomic DNA contaminations were removed by on column DNAse treatment with the RNase-free DNAse Set (Qiagen). RNA concentration and purity were measured with a NanoDrop spectrophotometer (ND-1000; Thermo Fisher Scientific Inc, Wilmington, DE). 1 µg total RNA was converted to cDNA using the RT Profiler First Strand Kit (SABiosciences, Frederick, MD) according to the manufacturer’s instructions.

### Quantitative PCR

The 84-gene PCR array RT^2^ Profiler PCR Array Human Angiogenesis (PAHS-024A) from SABiosciences was used for expression profiling of genes known to be involved in angiogenesis. 102 µl diluted cDNA was mixed with 1350 µl RT^2^ SYBR Green ROX qPCR Mastermix (Qiagen) and 1248 µl H_2_O, and 25 µl of this experimental cocktail was added to each well of the 96-well array plate. Real-time PCR was performed on an ABI 7900HT Fast Real-Time PCR instrument (Applied Biosystems, Carlsbad, CA). The cycling program consisted of one DNA polymerase activation cycle (10 min at 95°C) and 40 amplification cycles (15 s at 95°C followed by 1 min at 60°C). The threshold cycle (C_T_) for each well was calculated by using the instrument’s software. Each tumor line was run in three biological replicates. Data analysis was performed as recommended by the manufacturer. A C_T_-value of 33 (15 cycles above the positive PCR control) was defined as the detection limit of the system and, consequently, all C_T_-values above 33 were set to 33 in the analysis. The array included 5 endogenous control genes (housekeeping genes). Two of these genes, glyceraldehyde-3-phosphate dehydrogenase (GAPDH) and β-actin (ACTB), showed stable expression across 5 different melanoma lines (data not shown) and were chosen as normalization genes. Thus, each replicate C_T_-value was normalized to the mean C_T_ of GAPDH and ACTB (ΔC_T_  =  C_T_^gene of interest^ - C_T_^mean(GAPDH,ACTB)^). The normalized expression level of each gene was calculated from the 3 biological replicates as 2- mean ΔCT. Significantly expressed genes were defined as genes with mean C_T_ <33. Furthermore, genes were considered to have high expression if 2^- mean ΔCT^ >0.001 (corresponding to a C_T_-value of ∼26).

### Statistical Analysis

Correlations between parameters were searched for by linear regression analysis. Statistical comparisons of data were carried out by use of the Student’s *t*-test when the data complied with the conditions of normality and equal variance and otherwise by non-parametric analysis using the Mann-Whitney rank sum test. Probability values of P<0.05 were considered significant. Statistical analysis was performed with SigmaStat statistical software (SPSS, Chicago, IL).

## Results

### A-07-GFP and R-18-GFP Tumors Grew as Hemispheres

**Figure 1:**
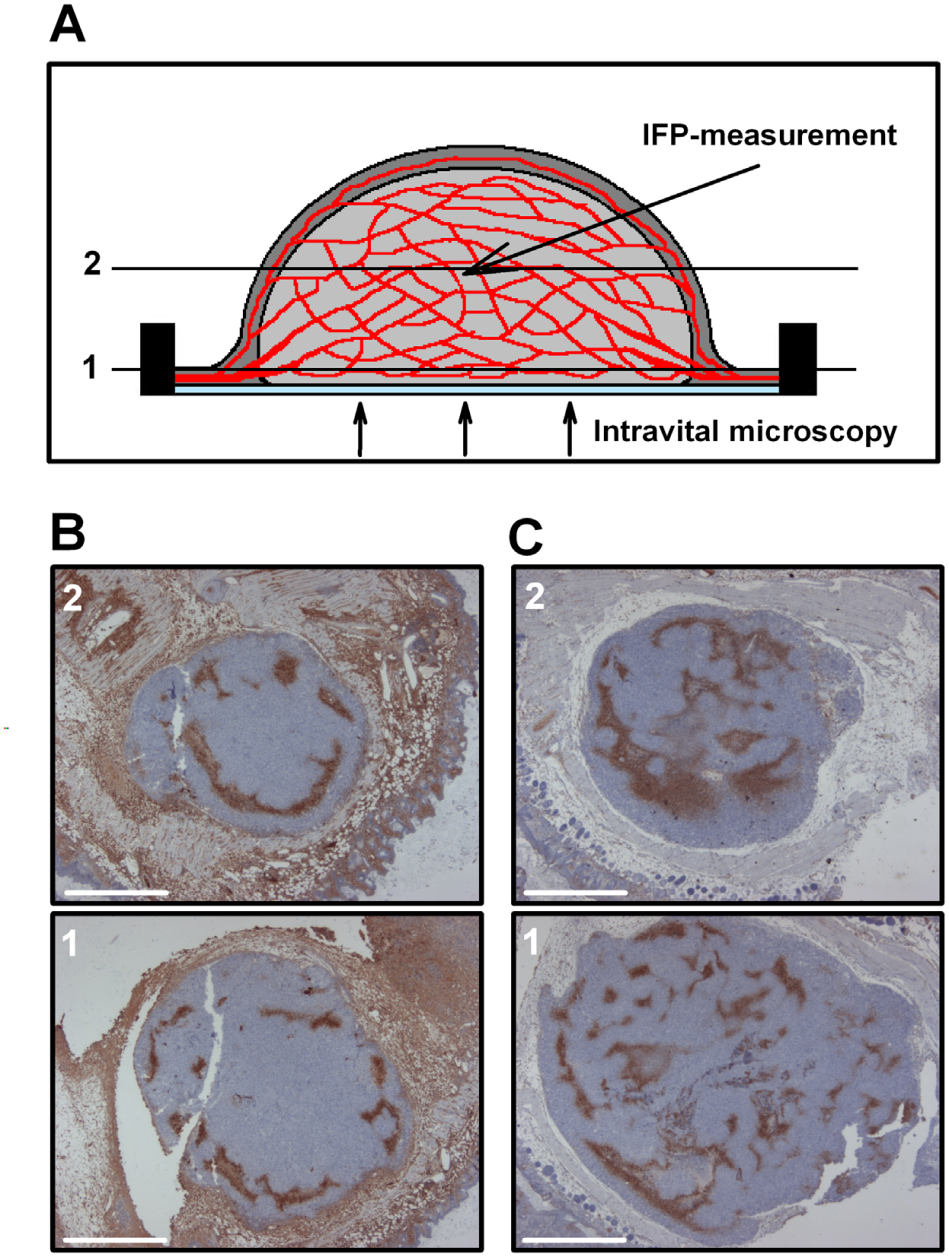
Growth and histology of window chamber tumors. **A**) Schematic illustration of a transversal section through a tumor growing in the dorsal skin fold window chamber. Dark grey areas represent normal tissue, light grey areas represent tumor tissue, red areas represent vasculature, and the light blue area represents the plastic window. Two planes parallel to the window are indicated. Plane 1 represents the plane imaged with intravital microscopy while plane 2 represents a plane through the tumor center, where IFP was measured. **B-C**) Histological sections stained for the hypoxia marker pimonidazole corresponding to the planes indicated in A) for R-18-GFP tumors showing peripheral staining (**B**) and scattered staining (**C**). Scale bars, 1 mm.

Thirteen A-07-GFP and 10 R-18-GFP tumors growing in dorsal window chambers were subjected to intravital microscopy examination and subsequent IFP measurement. The window chamber tumors grew as hemispheres surrounded by a plastic window and normal skin, as illustrated in Figure 1A. All tumors were highly vascularized, and the supplying arteries and the draining veins were located in the surrounding normal tissue within the chamber. Two different tumor planes parallel to the window are indicated in Figure 1A. Plane 1 represents the plane imaged with intravital microscopy, while plane 2 represents the tumor center, where IFP was measured. Pimonidazole-stained histological sections corresponding to these two planes are presented for two different R-18-GFP tumors in Figure 1B and 1C. Both tumors showed a similar hypoxia staining pattern in the two planes (Figure 1B-C, *upper vs. lower panels*). Moreover, the hypoxia staining patterns differed substantially between the two tumors, with peripheral staining in one tumor (Figure 1B), and scattered staining throughout the tumor in the other (Figure 1C).

### High IFP Correlated with Low Growth Rate in A-07-GFP Tumors and with High Growth Rate in R-18-GFP Tumors

**Figure 2:**
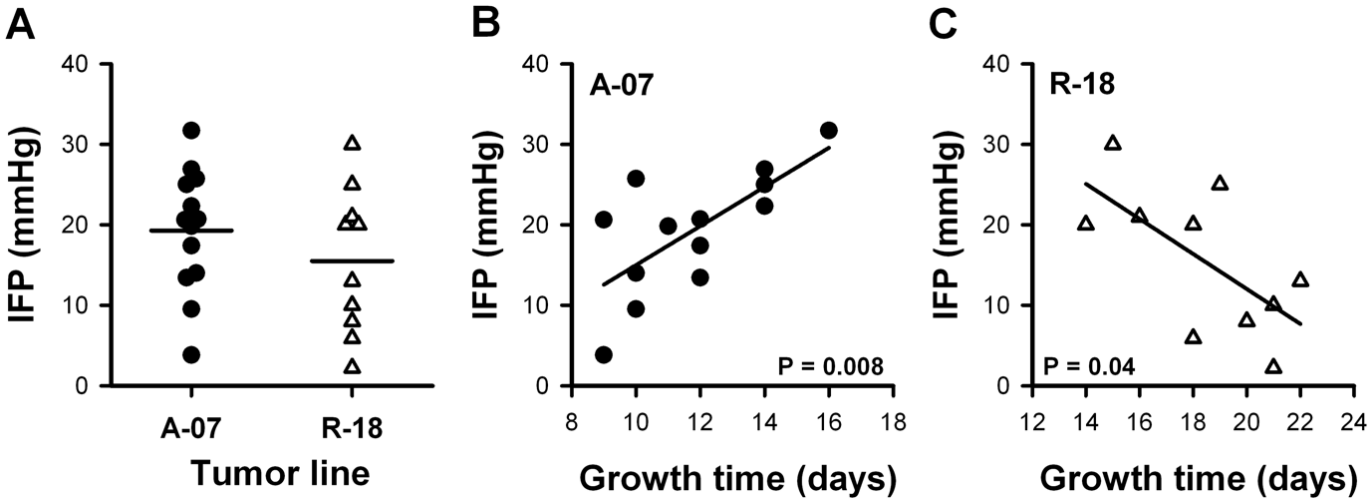
IFP and growth time in individual A-07-GFP and R-18-GFP tumors. **A**) IFP-values measured in the center of individual A-07-GFP and R-18-GFP tumors. **B–C**) IFP versus growth time for A-07-GFP (**B**) and R-18-GFP (**C**) tumors. Points represent individual A-07-GFP (•) and R-18-GFP (Δ) tumors. Horizontal bars represent mean values (A). Lines represent linear regression curves (B**–**C).

IFP in individual A-07-GFP tumors ranged from 4 to 32 mmHg with a mean value of 19.3 mmHg, whereas IFP in individual R-18-GFP tumors ranged from 2 to 30 mmHg with a mean value of 15.5 mmHg. There was no significant difference in IFP between A-07-GFP and R-18-GFP tumors (Figure 2A; P>0.05). The experiments were performed on the day the tumors had reached their maximum size within the chamber. This resulted in a variation in growth time that reflected the variation in growth rate between individual tumors. The growth time varied from 9 to 16 days for A-07-GFP tumors, and from 14 to 22 days for R-18-GFP tumors. High IFP was associated with low growth rate in A-07-GFP tumors. Thus, a positive correlation was found between IFP and growth time (Figure 2B; P = 0.008). Conversely, high IFP was associated with high growth rate in R-18-GFP tumors. Thus, an inverse correlation was found between IFP and growth time in R-18-GFP tumors (Figure 2C; P = 0.04).

### High IFP Correlated with Low Vascular Density in A-07-GFP Tumors and with High Vascular Density in R-18-GFP Tumors

**Figure 3:**
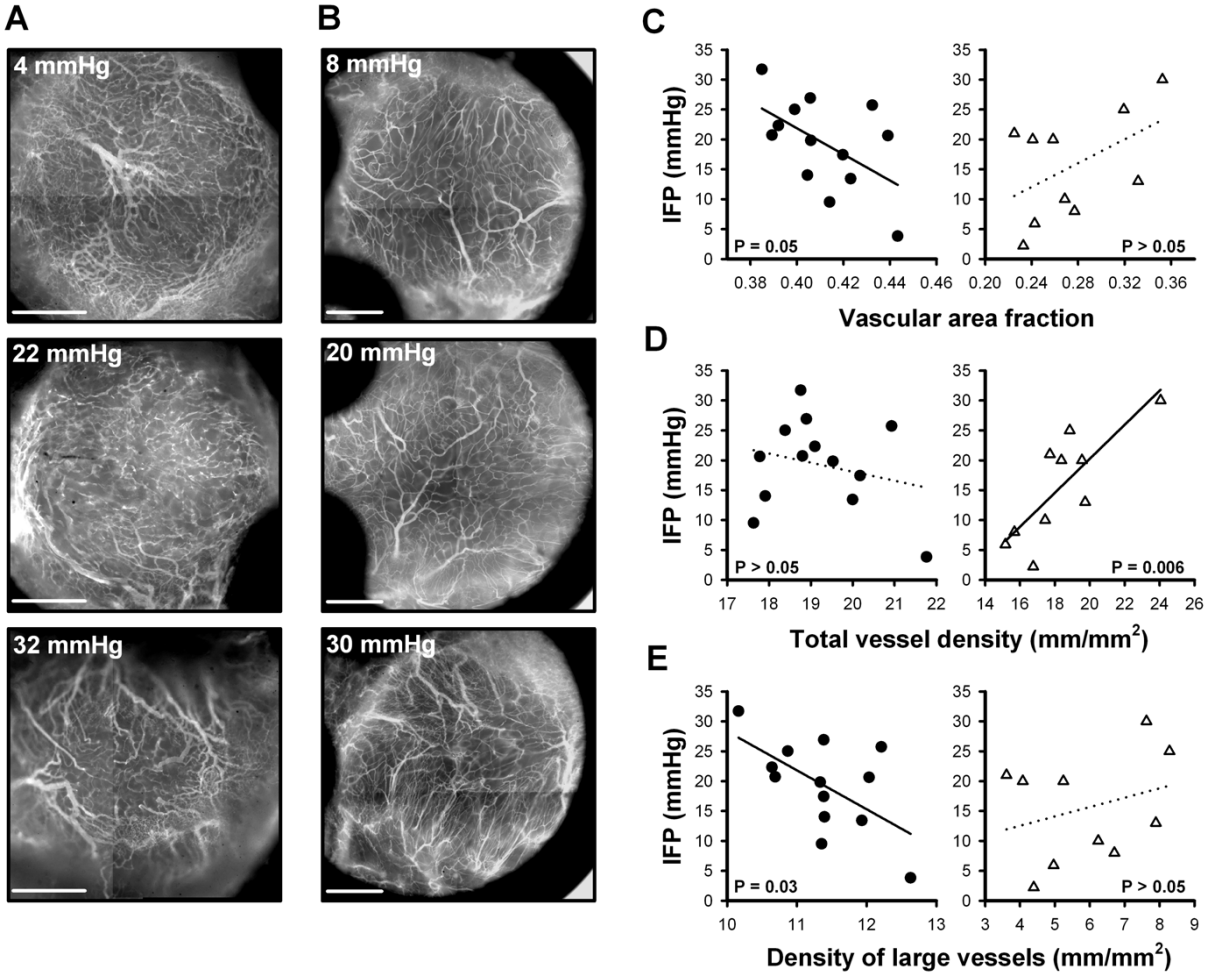
IFP and vascular density. Fluorescence images showing vascular networks of A-07-GFP (**A**) and R-18-GFP (**B**) tumors representing low (upper panel), intermediate (middle panel), and high (lower panel) IFP values. Scale bars, 1 mm. **C–E**) IFP versus vascular density calculated as vascular area fraction (**C**), total vessel density (**D**), and density of large vessels (density of vessels with diameter >20 µm) (**E**). Points represent individual A-07-GFP (•) and R-18-GFP (Δ) tumors. Lines represent linear regression curves. Solid lines, P<0.05; dotted lines, P>0.05.

Vascular networks of three A-07-GFP and three R-18-GFP tumors varying widely in IFP are shown in Figure 3A and 3B, respectively. Qualitative studies revealed a tendency for vascular density to decrease with increasing IFP in A-07-GFP tumors (Figure 3A) and to increase with increasing IFP in R-18-GFP tumors (Figure 3B). This was studied quantitatively by using three different parameters for vascular density: vascular area fraction, total vessel density, and density of large vessels (vessels with diameter >20 µm). The quantitative data were in accordance with the qualitative observations (Figure 3C-E). Thus, in A-07-GFP tumors, significant inverse correlations were found between IFP and vascular area fraction (Figure 3C, *left*; P = 0.05) and between IFP and density of large vessels (Figure 3E, *left*; P = 0.03). In R-18-GFP tumors, a significant positive correlation was found between IFP and total vessel density (Figure 3D, *right*; P = 0.006). IFP also tended to decrease with increasing total vessel density in A-07-GFP tumors and to increase with increasing vascular area fraction or density of large vessels in R-18-GFP tumors, although the correlations were not statistically significant (Figure 3C-E, dotted lines; P>0.05). Interestingly, small vessels contributed significantly to the positive correlation found between IFP and vascular density in R-18-GFP tumors (i.e. total vessel density was significantly correlated to IFP while density of large vessels was not). Conversely, only large vessels contributed to the inverse correlation between IFP and vascular density in A-07-GFP tumors (i.e. density of large vessels was significantly correlated to IFP while total vessel density was not).

### High IFP Correlated with High Plasma Velocity in Tumor Arterioles Relative to Tumor Venules

**Figure 4:**
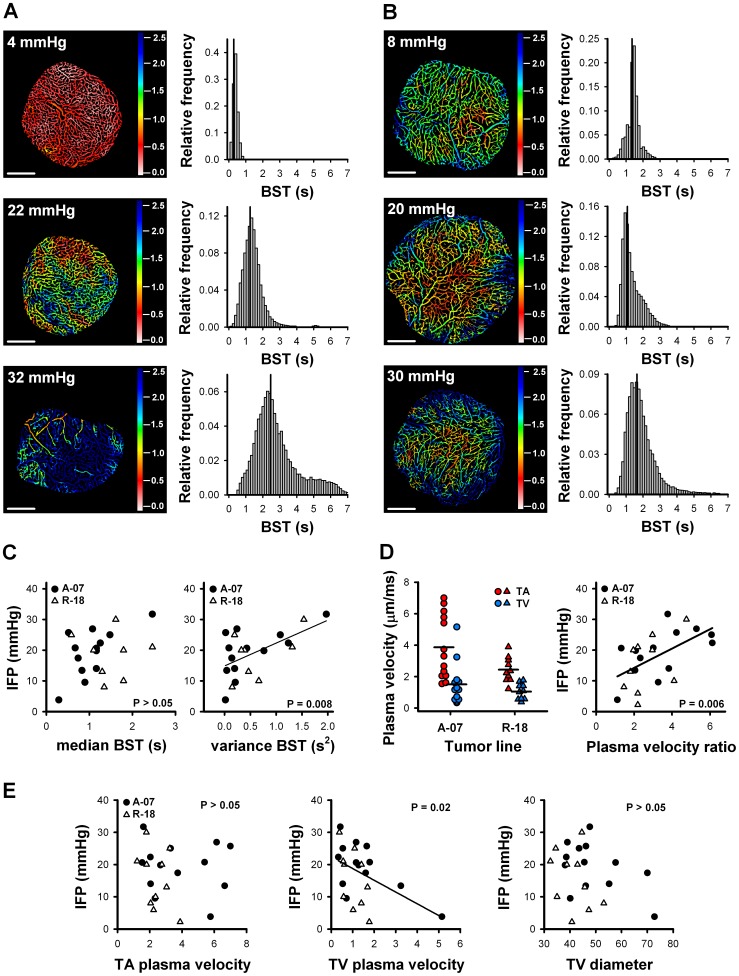
IFP and vascular function. Blood supply time (BST) images and BST frequency distributions of A-07-GFP (**A**) and R-18-GFP (**B**) tumors representing low (upper panel), intermediate (middle panel), and high (lower panel) IFP values. Scale bars, 1 mm; color bars, BST scale in seconds. Vertical lines in BST frequency distributions indicate median BST. **C**) IFP versus median BST (left) and variance BST (right). **D**) Plasma velocity in tumor arterioles (TAs; red symbols) and tumor venules (TVs; blue symbols), and IFP versus ratio of TA plasma velocity to TV plasma velocity. **E**) IFP versus TA plasma velocity (left panel), TV plasma velocity (middle panel), and TV diameter (right panel). Points represent individual A-07-GFP (•) and R-18-GFP (Δ) tumors. Horizontal bars represent mean values. Lines represent linear regression curves.

To study the relationship between IFP and tumor vasculature further, vascular function was quantified using the parameter BST. Figure 4A and 4B show BST images and BST frequency distributions of the same tumors as those illustrated in Figure 3A and 3B, respectively. BST increased with increasing IFP in A-07-GFP tumors (Figure 4A) but not in R-18-GFP tumors (Figure 4B). Moreover, the intratumor variation in BST increased with increasing IFP in both A-07-GFP and R-18-GFP tumors (Figure 4A and 4B). Quantitative analysis of the BST frequency distributions confirmed these observations (Figure 4C). Thus, median BST was significantly correlated to IFP in A-07-GFP tumors (P = 0.02) but not in R-18-GFP tumors (P>0.05). This resulted in no general correlation between IFP and median BST with all tumors pooled together (Figure 4C, *left*; P>0.05). On the other hand, a significant correlation was found between IFP and variance BST with all tumors pooled together (Figure 4C, *right*; P = 0.008).

High variance BST indicates that it takes a long time for the blood to pass from the tumor arterioles to the tumor venules. This time will depend on the total distance along tumor vessels, the blood flow velocity in the supplying arteries, and the decrease in blood flow velocity from the tumor arterioles to the tumor venules as a result of blood flow resistance in the tumor capillary network. We hypothesized that the positive correlation found between IFP and variance BST was a result of a larger decrease in blood flow velocity from the tumor arterioles to the tumor venules in the tumors with high IFP than in those with low IFP. To test this hypothesis, we measured the plasma velocity in tumor arterioles (TAs) and tumor venules (TVs) (Figure 4D, *left*). As expected, the plasma velocity decreased from the TAs to the TVs in all tumors. However, the ratio of TA velocity to TV velocity varied widely among individual tumors. Thus, the plasma velocity was up to 6-fold higher in the TAs than in the TVs in some tumors. There was a significant positive correlation between IFP and the ratio of TA plasma velocity to TV plasma velocity (Figure 4D, *right*; P  = 0.006). This correlation was not a consequence of differences on the arterial side of the tumor microvasculature, as there was no correlation between IFP and TA plasma velocity (Figure 4E, *left*; P>0.05). Furthermore, IFP correlated with TV plasma velocity (Figure 4E, *middle panel*; P = 0.02) but not with TV diameter (Figure 4E, *right*; P>0.05), suggesting that the blood flow resistance in the tumor capillary networks rather than the blood flow resistance in the tumor venules was associated with high IFP in A-07-GFP and R-18-GFP tumors.

### A-07-GFP Tumors with High IFP Showed High Vessel Tortuosity

**Figure 5:**
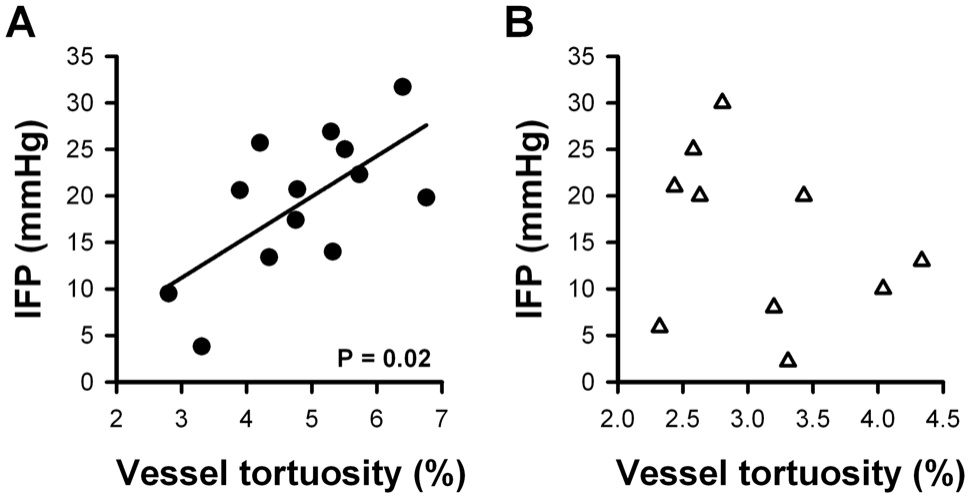
IFP and vessel tortuosity. IFP versus median vessel tortuosity in A-07-GFP (**A**) and R-18-GFP (**B**) tumors. Points represent individual A-07-GFP (•) and R-18-GFP (Δ) tumors. Lines represent linear regression curves.

The opposite correlations found between IFP and growth rate or vascular density in A-07-GFP and R-18-GFP tumors suggest that different vascular abnormalities may cause blood flow resistance and, hence, elevated IFP in the two melanoma lines. To search for possible mechanisms, vessel diameter, vessel segment length, and vessel tortuosity were quantified. No correlations were found between IFP and median vessel diameter or between IFP and median vessel segment length in either tumor line (P>0.05; data not shown). There was however a positive correlation between IFP and median vessel tortuosity in A-07-GFP tumors (Figure 5A; P = 0.02). High vessel tortuosity also correlated with long growth time and with low vascular area fraction in these tumors (P = 0.01 and P = 0.008 respectively; data not shown). Thus, A-07-GFP tumors with low growth rate, low vascular density, and high IFP showed highly tortuous vessels indicating high geometric resistance to blood flow. In contrast, there was no correlation between IFP and median vessel tortuosity in R-18-GFP tumors (Figure 5B; P>0.05).

### The Direction of Blood Flow in R-18-GFP Tumors was from the Center to the Periphery

**Figure 6:**
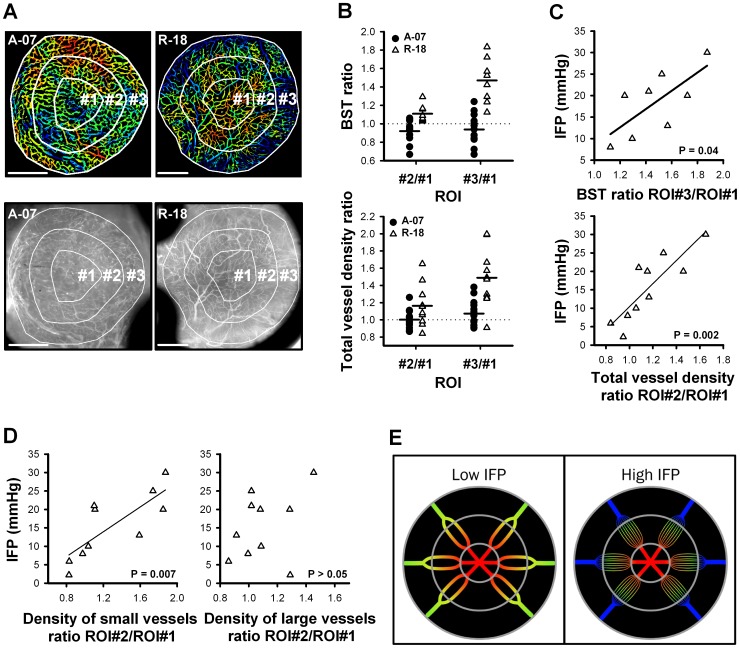
IFP and intratumor heterogeneity in R-18-GFP tumors. **A**) Illustration of concentric regions of interest: ROI#1, ROI#2, and ROI#3. Upper panel: BST-images. Lower panel: morphology images. **B**) Intertumor heterogeneity in BST (upper panel) and vascular density (lower panel) in A-07-GFP and R-18-GFP tumors calculated as median BST and total vessel density in ROI#2 and in ROI#3 relative to ROI#1 (ratio ROI#2/ROI#1 and ratio ROI#3/ROI#1). **C**) IFP versus BST ratio ROI#3/ROI#1 and IFP versus total vessel density ratio ROI#2/ROI#1 in R-18-GFP tumors. **D**) Vessel density ratio ROI#2/ROI#1 for small vessels (diameter <20 µm) and for large vessels (diameter >20 µm) in R-18-GFP tumors. Points represent individual A-07-GFP (•) and R-18-GFP (Δ) tumors. Horizontal bars represent mean values. Lines represent linear regression curves. **E**) Schematic illustration of blood flow pattern and vessel distribution in R-18-GFP tumors with low IFP and R-18-GFP tumors with high IFP. Red color indicates early blood supply; blue color indicates late blood supply.

To search further for possible mechanisms causing high blood flow resistance in A-07-GFP and R-18-GFP tumors, intratumor heterogeneity in BST and vascular morphology was investigated. Qualitative image analysis revealed large intratumor heterogeneity in both A-07-GFP and R-18-GFP tumors. Moreover, R-18-GFP tumors showed a systematic radial variation in vascular density and BST (Figure 3B and 4B) that was not observed in A-07-GFP tumors (Figure 3A and 4A). To quantify this radial variation, all tumors were subdivided into three concentric regions of interest (ROIs), as illustrated in Figure 6A. Median BST and total vessel density were then calculated in the central tumor region (ROI#1), the middle tumor region (ROI#2), and the peripheral tumor region (ROI#3). The radial variation in BST within each tumor was quantified by calculating the ratio of median BST in the middle and peripheral tumor regions relative to the central tumor region (ratio ROI#2/ROI#1 and ratio ROI#3/ROI#1, respectively). These ratios are shown in the upper panel of Figure 6B. In all R-18-GFP tumors, BST increased gradually from the center to the periphery. Thus, the BST ratios were >1, and the BST ratio ROI#3/ROI#1 was higher than the BST ratio ROI#2/ROI#1 in all R-18-GFP tumors. In accordance with qualitative observations of the imaging movies, this shows that the direction of blood flow in all R-18-GFP tumors was from the center to the periphery. The radial variation in total vessel density was calculated in the same way as radial variation in BST (Figure 6B, *lower panel*). In most R-18-GFP tumors, vascular density increased from the center to the periphery. Thus, total vessel density ratio ROI#2/ROI#1 and total vessel density ratio ROI#3/ROI#1 were >1 in most R-18-GFP tumors. In contrast to R-18-GFP tumors, A-07-GFP tumors showed no systematic radial blood flow pattern and no systematic radial variation in vessel density (Figure 6B).

### R-18-GFP Tumors with High IFP Showed Late Blood Supply and High Vessel Density in the Middle and Peripheral Tumor Regions Relative to the Tumor Center

The BST images presented in Figure 4B show that the radial variation in BST was more pronounced in R-18-GFP tumors with high IFP than in R-18-GFP tumors with low IFP. Accordingly, a positive correlation was found between IFP and BST ratio ROI#3/ROI#1 (Figure 6C, *upper panel*; P = 0.04). Similarly, the vascular morphology images presented in Figure 3B show that the radial variation in vascular density was more pronounced in R-18-GFP tumors with high IFP than in R-18-GFP tumors with low IFP. A strong positive correlation was found between IFP and total vessel density ratio ROI#2/ROI#1 (Figure 6C, *lower panel*; P = 0.002). The vessel density ratio ROI#2/ROI#1was also calculated for small vessels (vessels with diameter <20 µm) and large vessels (vessels with diameter >20 µm) separately. A significant positive correlation was found between IFP and vessel density ratio ROI#2/ROI#1 for small vessels (Figure 6D, *left*; P = 0.007), whereas no significant correlation was found for large vessels (Figure 6D, *right*; P>0.05).

In R-18-GFP tumors, most tumor arterioles were located in ROI#1, most tumor capillaries in ROI#2, and most tumor venules in ROI#3. Thus, the blood flow from ROI#1 to ROI#2 represents blood flow from the tumor arterioles to the tumor capillary network. The correlations found in Figure 6C suggest that the reduction in blood flow velocity and, hence, the blood flow resistance in the tumor capillaries of ROI#2 was decisive for IFP. A schematic illustration of the blood flow pattern and vessel distribution of R-18-GFP tumors with low IFP and R-18-GFP tumors with high IFP is presented in Figure 6E. The tumors with low IFP show blood flowing from the central tumor arterioles to a low number of medium-sized tumor capillaries. The tumors with high IFP show blood flowing from the central tumor arterioles to a high number of narrow tumor capillaries. This results in delayed blood supply in the peripheral tumor venules indicating high blood flow resistance in the capillary network.

### A-07 and R-18 Cells Showed Highly Different Angiogenic Profiles

**Figure 7:**
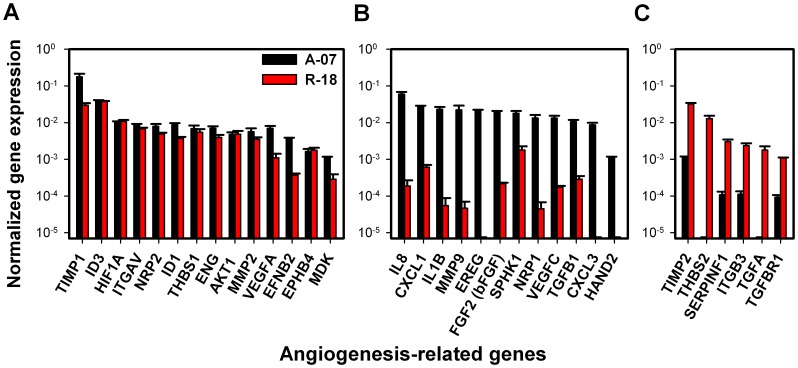
Angiogenic profiles of A-07-GFP and R-18-GFP cells. Normalized expression of angiogenesis-related genes with high expression in A-07-GFP and R-18-GFP cells (**A**), more than 10-fold higher expression in A-07-GFP than in R-18-GFP cells (**B**), and more than 10-fold higher expression in R-18-GFP than in A-07-GFP cells (**C**). Columns represent means of 3 biological replicates. Bars represent SEM. Gene expression was measured with quantitative PCR and normalized to housekeeping genes with stable expression (GAPDH and ACTB).

To investigate possible differences between the angiogenic profiles of A-07-GFP and R-18-GFP tumors, the expression of 84 known angiogenesis-related genes was quantified in A-07-GFP and R-18-GFP cells using a commercial quantitative PCR array. Of the 84 genes, 63 (75%) showed significant expression and 32 (38%) showed high expression in one or both melanoma lines. The normalized expression levels of the highly expressed genes are presented in Figure 7. Fourteen genes showed similar expression levels in the two melanoma lines (i.e. fold difference <10; Figure 7A), 12 genes showed more than 10-fold higher expression in A-07-GFP than in R-18-GFP cells (Figure 7B), and 6 genes showed more than 10-fold higher expression in R-18-GFP than in A-07-GFP cells (Figure 7C).

## Discussion

Vascular abnormalities associated with elevated IFP were studied by using A-07-GFP and R-18-GFP human melanomas growing in dorsal skin fold chambers as preclinical tumor models. These two melanoma lines were chosen for several reasons. A-07 and R-18 tumors grown intradermally in mice have been shown to retain several biological characteristics of the donor patient’s tumors, including angiogenic potential, histopathological appearance, radiation sensitivity, and metastatic pattern [31]. Furthermore, high intertumor heterogeneity in IFP has been demonstrated in xenografted tumors of both melanoma lines [12], [39]. Finally, A-07 and R-18 tumors have been shown to differ significantly in vascular parameters. Thus, A-07 tumors showed higher rate of angiogenesis, higher microvascular density, and higher perfusion rates than R-18 tumors [40].

It has been suggested that window chamber tumors growing as thin sheets between two windows may show higher IFP than three-dimensional tumors as a result of restrictions in growth and fluid flow [33], [41]. The tumors in the present study grew in a hemisphere shape in chambers with one single window, thus allowing high resolution imaging of vascular function and morphology in three-dimensional tumors that were accessible for IFP measurement without window removal. In this window chamber model, IFP was measured in the center of the tumors while the vascular parameters were measured in a plane close to the window. The tumor region defined as tumor center in the intravital microscopy images was located far from the supplying arteries and the draining veins in the normal tissue. The vasculature in this region was therefore expected to be representative of the vasculature in the tumor center rather than the tumor rim. Consequently, the vascular parameters measured close to the window were expected to be highly relevant for the IFP measured in the tumor center. The present data support this assumption. Thus, the hypoxia staining pattern in a plane close to the window was representative of the hypoxia staining pattern in a plane through the center of the tumor. Moreover, significant correlations were found between IFP and vascular parameters in both A-07-GFP and R-18-GFP tumors. The present data also show that differences in biological characteristics between A-07 and R-18 tumors were retained in the window chamber model, including growth rate and vascular density [31]. Furthermore, the IFP values measured in the A-07-GFP and R-18-GFP tumors in the present study were within the range of IFP values measured previously in small intradermal A-07 and R-18 tumors [12]. Consequently, A-07-GFP and R-18-GFP window chamber tumors were considered well-suited models for studying the association between IFP and vascular parameters.

Some tumors are characterized by microvessels with high resistance to transcapillary fluid flow, and the IFP of these tumors is expected to be lower than the microvascular hydrostatic pressure. IFP-values lower than the microvascular hydrostatic pressure has been demonstrated in experimental tumors [21], and in these tumors IFP is not necessarily correlated to morphological parameters governing the geometric resistance to blood flow. Most tumors however, are characterized by microvessels with low resistance to transcapillary fluid flow [42], [43]. Theoretical and experimental studies have demonstrated that in this case, IFP is governed primarily by the microvascular hydrostatic pressure [7], [18], [24], [44]. Consequently, intertumor heterogeneity in IFP is expected to reflect intertumor heterogeneity in geometric and/or viscous resistance to blood flow in the microvascular networks of these tumors [1].

The microvessels of xenografted A-07 and R-18 tumors are characterized by incomplete endothelial lining, interrupted or absent basement membrane, and high vascular permeability to macromolecules [31], [45]. A-07-GFP and R-18-GFP tumors are thus representative of tumors showing low resistance to transcapillary fluid flow. Consequently, even though microvascular hydrostatic pressure was not measured in the present study, it is highly reasonable to expect that IFP was determined primarily by the microvascular hydrostatic pressure and, hence, the microvascular blood flow resistance in these two melanoma lines. The present study is consistent with this assumption. Thus, high IFP was associated with high vessel tortuosity in A-07-GFP tumors and with high density of narrow tumor capillaries in R-18-GFP tumors. Vascular networks characterized by high vessel tortuosity or by low vessel diameters show high geometric resistance to blood flow, high microvascular pressure, and low blood flow velocities [15], [16]. Accordingly, a large decrease in blood flow velocity from the tumor arterioles to the tumor venules was detected in A-07-GFP and R-18-GFP tumors with high IFP.

The vascular abnormalities that caused elevated IFP in A-07-GFP and R-18-GFP tumors were tumor-line specific. Moreover, the vascular architecture and blood flow patterns were distinctly different in A-07-GFP and R-18-GFP tumors. The present study further shows that these melanoma lines may differ substantially in angiogenic profile. Thus, of the 32 angiogenesis-related genes identified to have high expression in one or both melanoma lines, 38% showed more than 10-fold higher expression in A-07-GFP than in R-18-GFP cells, while 19% showed more than 10-fold higher expression in R-18-GFP than in A-07-GFP cells. The genes with higher expression in A-07-GFP cells included genes encoding for the pro-angiogenic factors interleukin-8 (IL-8) and basic fibroblast growth factor (bFGF). We have previously shown that the secretion rates of IL-8 and bFGF are significantly higher for A-07 than for R-18 cells, and that treatment with blocking antibodies against these factors significantly reduced angiogenic activity in A-07 but not R-18 tumors [46]. Consequently, it is likely that the tumor-line specific vascular abnormalities associated with high IFP in the present study were results of angiogenic activity caused by different angiogenic factors in the two melanoma lines.

The data reported here show that high IFP was associated with low growth rate and low vascular density in A-07-GFP tumors, and with high growth rate and high vascular density in R-18-GFP tumors. We have previously shown that tumor growth was closely related to angiogenic activity in a study of 4 melanoma lines, including A-07 and R-18 [31]. This relationship was confirmed in a more recent study including a total of 9 melanoma lines [47]. Therefore, it is likely that the combination of low growth rate and low vascular density observed in A-07-GFP tumors with high IFP indicated low angiogenic activity, while the combination of high growth rate and high vascular density observed in R-18-GFP tumors with high IFP indicated high angiogenic activity. As the vascular abnormalities associated with high IFP were distinctively different in the two melanoma lines, it is conceivable that they were results of high angiogenic activity in one melanoma line and of low angiogenic activity in the other. This is consistent with the suggestion that different angiogenic factors controlled angiogenesis in A-07-GFP and R-18-GFP tumors. Furthermore, the present data are consistent with previous studies in our lab. Thus, high IFP has previously been shown to correlate with low vessel density in A-07-GFP window chamber tumors [11]. Moreover, high IFP has been associated with high concentration of VEGF-A in small intradermal R-18 tumors [12]. As angiogenesis in R-18 tumors has been shown to depend on VEGF-A [46], R-18 tumors with high concentration of VEGF-A are expected to show higher angiogenic activity than R-18 tumors with low concentration of VEGF-A.

In order to develop effective strategies for lowering tumor IFP and, consequently, improve patient outcome, a thorough understanding of the basic mechanisms of elevated IFP in tumors is essential. The present study suggests that different angiogenic factors may be responsible for vascular abnormalities leading to high IFP in different human tumors of the same histological type. Strategies to normalize the tumor vasculature with anti-angiogenic agents should therefore most likely be individualized based on the angiogenic profile of the tumor. In order to individualize treatment and monitor treatment effect, reliable and non-invasive biomarkers for IFP are highly warranted. Different vascular parameters have been proposed as potential biomarkers, including blood perfusion and relative tumor blood volume measured by dynamic contrast-enhanced MRI [39], [48]. The present study suggests that these vascular parameters may have limited potential as clinical biomarkers for IFP, as tumors derived from two different melanoma lines demonstrated opposite relationships between IFP and vascular density.

In summary, high IFP was associated with high geometric resistance to blood flow caused by tumor-line specific vascular abnormalities in xenografted tumors from two human melanoma lines with different angiogenic profiles. The vascular abnormalities responsible for high IFP were associated with high angiogenic activity in one melanoma line, and with low angiogenic activity in the other. These observations show that the relationship between tumor angiogenesis and IFP is complex and may differ substantially among human tumors of the same histological type.
